# Outcome of intra-arterial thrombolysis in patients with diabetes and acute lower limb ischemia: a propensity score adjusted analysis

**DOI:** 10.1007/s11239-017-1563-4

**Published:** 2017-10-04

**Authors:** Talha Butt, Anders Gottsäter, Jan Apelqvist, Gunnar Engström, Stefan Acosta

**Affiliations:** 1 Department of Clinical Sciences, Lund University, Ruth Lundskogsg 10, 205 02 Malmö, Sweden; 2 Vascular Centre, Department of Cardio-Thoracic and Vascular Surgery, Skåne University Hospital, Malmö, Sweden; 3 Department of Endocrinology, Skåne University Hospital, Malmö, Sweden

**Keywords:** Acute lower limb ischemia, Diabetes mellitus, Propensity score adjusted analysis, Thrombolysis

## Abstract

The presence of diabetes mellitus is rarely addressed in acute lower limb ischaemia (ALLI). The aim of this study was to evaluate the outcome of local intra-arterial thrombolysis for ALLI in patients with diabetes mellitus (DM). Outcome of all thrombolytic events performed in an endovascular first-strategy centre during a 13-year period between 2001 and 2013 in patients with ALLI were followed to January 2017. A propensity score adjusted analysis was performed to evaluate results in patients with (n = 83) versus without (n = 316) DM. Patients with DM were younger (p = 0.001), more often women (p = 0.014), more often had renal insufficiency (p = 0.041), foot ulcers (p < 0.001), and thrombosis (p = 0.032) than the patients without DM. At presentation, patients with DM had a lower degree of ischemia judged by the Rutherford classification, compared to those without DM (p = 0.023). None of the 83 diabetic patients had a popliteal artery aneurysm, compared to 25 (7.9%) of the 316 patients without DM (p = 0.008). The amount of tPA administered to patients with DM was higher than to patients without DM (p = 0.03). In the propensity score adjusted analysis, patients with DM had a higher rate of major amputation at 1 (OR 2.52; 95% CI 1.22–5.20) and 3 years (OR 2.52; 95% CI 1.26–5.04), and a lower amputation-free survival at 3 years (OR 0.46; 95% CI 0.25–0.85), than those without DM. Patients with DM presenting with ALLI differ in clinical characteristics, presentation, and aetiology compared to patients with DM, and have a higher rate of major amputation and lower amputation-free survival rate after intra-arterial thrombolysis.

## Introduction

Local intra-arterial thrombolysis is the preferred treatment option for acute lower limb ischaemia (ALLI) in many vascular centres. As logistics, imaging, technique, material, and learning has progressed rapidly towards improved performance of endovascular therapy, the proportion of work load for open vascular surgery in ALLI has decreased. Certainly, revascularization can be achieved swiftly by thrombolysis, especially in combination with perioperative aspiration and mechanical thrombectomy [1].

Several factors have been associated with improved or adverse outcome in terms of amputation-free survival in ALLI patients undergoing thrombolysis. In a recent report on outcomes after thrombolysis for ALLI, end-stage renal disease and poor pedal outflow were predictors for limb loss after multi-variable testing, whereas diabetes mellitus (DM) and the Rutherford Classification were not [2]. The impact of the presence of DM in a large proportion of ALLI patients; for clinical presentation, aetiology, results of thrombolysis, and outcome has not been addressed sufficiently. It is well-known that patients with DM has a more extensive distal arterial occlusive disease with a high prevalence of long tibial occlusions compared to nondiabetic patients [3], and more often suffer from other late manifestations such as nephropathy, neuropathy, and retinopathy which may influence clinical presentation and treatment outcomes [4].

The vascular centre at Skåne University Hospital, Malmö, Sweden, has for decades been an established endovascular first-strategy centre. The main aim of the present study was to evaluate the impact of DM on amputation-free survival in patients undergoing thrombolysis for ALLI in a propensity score adjusted analysis in a large cohort of patients.

## Methods

### Setting

Skåne University Hospital is the third largest hospital in Sweden, located in Malmö and Lund. Both cities together have a population of about 445,000 inhabitants and the primary catchment population is 800,000 inhabitants. The Centre does not only serve these patients but also acts as a referral centre for the southern part of Sweden. This study was approved by the research ethical review board and the study protocol conformed to the ethical guidelines of the 1975 Declaration of Helsinki.

### Study population

All thrombolytic treatments performed in patients with ALLI between January 1st 2001 and December 31st 2013 were included. Patients undergoing intra-arterial thrombolysis between 2001 and 2010 have been reported previously with a follow up until January 17, 2012 [5]. No previous analysis has been performed with respect to groups with or without DM.

### Thrombolysis

Before thrombolysis treatment is begun, several conditions must be met. Absolute contraindications are described in a local memo; operation or organ biopsy ≤ 2 weeks, cerebral infarction ≤ 6 weeks, cerebral metastasis, known arteriovenous cerebral malformations and epidural catheter or puncture of the dura ≤ 3 days. If none of these absolute contraindications are present several blood tests are performed including: creatinine, hemoglobin, aspartate aminotransferase, alanine aminotransferase, bilirubin, lactate dehydrogenase, activated partial thromboplastin time (APTT), prothrombin complex, and platelet count.

Preferably a contralateral puncture is made in the common femoral artery. Catheterization is usually made through the occlusion, and sometimes complementary high dose thrombolysis (pulse spray), aspiration and mechanical micro fragmentation is performed to shorten treatment time. A thrombolysis catheter with side holes in a 5–20 cm segment is placed in the occlusion and the lytic agent, alteplase, a recombinant tissue plasminogen activator, is deposited. A heparin bolus of 5000 IE is given at start of the procedure, followed by continuous heparin infusion monitored by regular APTT every 6 h to achieve an APTT of 60–90 s during therapy.

### Definitions

Degree of ischemia was defined at admission according to the Rutherford classification [6]. Diabetes mellitus was considered present in patients on antidiabetic therapy with diet, oral hypoglycemic agents, or insulin.

Degree of lysis was defined as complete, partial, lysis but no run-off, or no lysis [7].

Run off after lysis was determined by using angiographic images evaluated at both the beginning and end of the thrombolytic procedure. Major amputation was defined as amputation above foot-level. Primary patency was defined as patent initial intervention and no need for additional intervention, and secondary patency as need of an additional intervention after complete occlusion of the first procedure. Ischemic heart disease was defined as a history of myocardial infarction, angina pectoris, coronary artery bypass, or percutaneous coronary angioplasty. Patients with a history of stroke (cerebral bleeding or infarction) or transient ischemic attack were considered to have cerebrovascular disease. Anemia was considered when hemoglobin (Hb) levels was below 134 g/L in men and 117 g/L in women. Renal insufficiency was considered present when serum creatinine level was higher than 105 μmol/L in men and 90 μmol/L in women.

### Follow-up

All patients were followed from the time of inclusion to amputation or death, or to end of-follow up January 5th 2017. Information on amputations was retrieved from medical charts, and survival was followed-up by linkage to the National Population Registry.

### Statistical methods

Differences in proportions or continuous variables were analysed with Pearson’s chi square test and Mann Whitney U test, respectively. The association between female gender and amputation at three years were assessed with multi-variable regression analysis, with entry of age and DM as covariates. Multivariate adjustments by logistic regression is limited by the number of endpoints, and a limited number of covariates should be modelled [8]. We therefore chose a propensity score technique to adjust for multiple risk factors [9, 10]. With this method, several risk factor for adverse outcome are used to calculate a propensity score, reflecting the differences in risk factors between exposed and unexposed individuals, i.e., those with and without diabetes. In the next step, the propensity score can be used to adjust for differences between diabetes and non-diabetes in an analysis of outcomes. In the first step, we used a logistic regression model, with sex, age, IHD, renal insufficiency, atrial fibrillation, presence of foot ulcer, Rutherford classification, thrombosis, amount of t-PA, and treatment with clopidogrel as independent variables, and diabetes as the dependent variable. All p values < 0.05 were considered statistically significant.

## Results

### Characteristics of diabetic and non diabetic patients

Patients with DM were younger (p = 0.001), more often had renal insufficiency (p = 0.041), and foot ulcers (p < 0.001) than those without DM (Table 1). Diabetic patients had a lower degree of ischemia according to Rutherford class, compared to non-diabetic patients (p = 0.023) (Table 2).

**Table 1:** Comparative factors in thrombolysis for acute lower limb ischaemia performed in patients with and without diabetes mellitus (DM)

Total	All (%)	With DM (%)	Without DM (%)	p value	OR (95%CI)
Women	181	48 (57.8)	133 (42.1)	0.014	
Age (median [IQR])	71 (63–78)	60 (60–73)	72 (64–79)	0.001	
Symptom duration (h)	48 (24 − 20)	48 (19–134)	48 (24–120)	0.78	
CRP (mg/L)	11 (5–34)	9 (5–37)	5 (11–34)	0.53	
Comorbidities					
Ischemic heart disease n = 399	132 (33.1)	34 (41.0)	98 (31.0)	0.086	1.54 (0.9–2.5)
Cerebrovascular disease n = 399	60 (15.0)	12 (14.5)	48 (15.2)	0.868	0.9 (0.5–1.9)
Anemia n = 387	104 (26.9)	25 (32.5)	79 (25.5)	0.216	1.4 (0.8–2.4)
Renal insufficiency n = 393	123 (31.3)	33 (40.7)	90 (28.8)	0.040	1.7 (1.0-2.8)
Atrial fibrillation n = 399	98 (24.6)	14 (16.9)	84 (26.6)	0.067	0.6 (0.3-1.0)
Presence of foot ulcer	46 (11.5)	20 (24.4)	26 (8.3)	< 0.001	3.6 (1.9–6.8)
Medications at admission					
Aspirin n = 398	225 (56.5)	46 (56.5)	179 (56.6)	0.929	1.0 (0.6–1.6)
Clopidogrel n = 398	42 (10.6)	15 (18.3)	27 (8.5)	0.010	2.4 (1.2–4.8)
Warfarin n = 399	51 (12.8)	13 (15.7)	38 (12.0)	0.377	1.4 (0.7–2.7)

**Table 2:** Etiology and indication of thrombolysis for acute lower limb ischaemia performed in patients with and without diabetes mellitus (DM)

	All (%)	With DM (%)	Without DM (%)	p value	OR (95% CI)
Total	399	83 (20.8)	316 (79.2)		
Degree of ischemia—Rutherford Class (n = 396)					
I	65 (16.4)	23 (28.0)	42 (13.4)		
IIa	231 (58.3)	42 (51.2)	189 (60.2)		
IIb	99 (25.0)	16 (19.5)	83 (26.4)		
III	1 (0.3)	1 (1.2)	0 (0.0)	0.023	
Etiology (n = 399)					
Native artery occlusion	200 (50.1)	40 (48.2)	160 (50.6)	0.69	0.9 (0.6–1.5)
Bypass occlusion (all)	117 (29.3)	27 (32.5)	90 (28.5)	0.90	1.2 (0.7–2.0)
Vein bypass occlusion	23 (5.8)	5 (6.0)	18 (5.7)	0.91	1.1 (0.4–2.9)
Synthetic bypass occlusion	94 (23.6)	22 (26.5)	72 (22.8)	0.48	1.2 (0.7–2.1)
Endoprosthesis occlusion	82 (20.6)	16 (19.3)	66 (20.9)	0.75	0.9 (0.5–1.7)
Indication (n = 399)					
Thrombosis	94 (23.6)	27 (32.5)	67 (21.2)	0.032	1.8 (1.1–3.1)
Embolus	81 (20.3)	13 (15.7)	68 (21.5)	0.24	0.7 (0.4–1.3)
Popliteal artery aneurysm	25 (6.3)	0 (0.0)	25 (7.9)	0.008	–
Graft/Stent occlusion	199 (49.9)	43 (51.8)	156 (49.4)	0.69	1.1 (0.7–1.8)

### Aetiology and indication for thrombolysis in diabetic and non diabetic patients

Thrombotic occlusion was more common (p = 0.032) in diabetic patients (Table 2). None of the 83 patients with DM had a popliteal artery aneurysm compared to 25 (7.9%) of the 316 patients without diabetes (p = 0.008).

### Effects of thrombolysis in diabetic and non diabetic patients

The amount of tPA administered in diabetic patients was higher compared to in those without diabetes (p = 0.03) (Table 3). There were trends towards lower primary patency rate at 3 years (OR 0.6; 95% CI 0.4–1.1), higher re-intervention rate (OR 1.4; 95% CI 1.0–1.9), and lower secondary patency at one (OR 0.5; 95% CI 0.3–0.9) and 3 years (OR 0.5; 95% CI 0.3–0.8) in patients with diabetes compared to those without.

**Table 3:** Characteristics of thrombolysis for acute lower limb ischaemia performed in patients with and without diabetes mellitus (DM)

	All (%)	With DM (%)	Without DM (%)	p value	OR (95%CI)
Total	399	83 (20.8)	316 (79.2)		
Degree of lysis (n = 399)					
Complete lysis	162 (40.9)	38 (45.8)	124 (39.6)		
Partial lysis	178 (44.9)	32 (38.6)	146 (46.6)		
Lysis, but no run-off	17 (4.3)	4 (4.8)	13 (4.2)		
No lysis	38 (9.6)	9 (10.8)	29 (9.3)	0.53	
Duration of lysis (h)	21 (17–32)	22 (18–36)	21 (17–31)	0.27	
Amount of t-PA (mg)	21 (15–30)	25 (18–35)	21 (15–29)	0.03	
Adjuvant revascularization	320 (80.2)	69 (83.1)	251 (79.4)	0.45	1.3 (0.7–2.4)
Endovascular	252	58	194	0.16	1.5 (0.9–2.5)
Open	28	4	24	0.38	0.8 (0.5–1.4)
Hybrid	40	7	33	0.59	0.9 (0.7–1.2)
Major bleeding (n = 399)	65 (16.3)	11 (13.3)	54 (17.1)	0.40	0.7 (0.4–1.5)
Fasciotomy (n = 399)	23 (5.8)	4 (4.8)	19 (6.0)	0.678	0.8 (0.3–2.4)

### Major amputation

The overall 1-year and 3 year major amputation rate was 18% (72/399) and 23% (92/399), respectively. The major amputation rate at one (29% [24/83] versus 15% [48/316]; p = 0.004) and three (36% [30/83] versus 20% [62/316]; p = 0.001) years was higher among diabetic patients compared to non-diabetics. After adjustment for age and DM, women tended (p = 0.059) to have a higher rate of major amputation at three years (52/181; 28.7%) compared to men (40/218; 18.3%). Patients with DM and foot ulcer had a higher major amputation rate at one (50% [10/20] versus 23% [14/62]; p = 0.019) and three (65% [13/20] versus 27% [17/62]; p = 0.002) years compared to those with DM and no foot ulcer.

### Propensity score adjusted analysis

In the propensity score adjusted analysis, patients with DM had a higher rate of major amputation at 1 (OR 2.52; 95% CI 1.22–5.20) and 3 years (OR 2.52; 95% CI 1.26–5.04), and a lower amputation-free survival at 3 years (OR 0.46; 95% CI 0.25–0.85) compared to those without diabetes (Table 4).

**Table 4:** Propensity score adjusted analysis in patients with and without diabetes mellitus undergoing thrombolysis for acute lower limb ischaemia

	OR (95% CI)
Amputation	
30 day	1.61 (0.60–4.31)
1 year	2.52 (1.22–5.2)
3 year	2.52 (1.26–5.04)
Death	
30 days	2.60 (0.33–20.58)
1 year	1.12 (0.44–2.86)
3 year	1.46 (0.74–2.87)
Amputation free survival	
30 days	0.95 (0.37–2.45)
1 year	0.53 (0.28–1.02)
3 year	0.46 (0.25–0.85)

## Discussion

Patients with DM comprised more than one-fifth of all patients treated for ALLI in the present population-based study. The presence of DM is often associated with a spectrum of subclinical or manifest comorbidities different from classical atherosclerotic disease, and therefore merits a separate analysis. The predilection for multiple crural vessel involvement combined with extensive arterial calcification and long occlusions increases the technical challenges associated with revascularization [11]. Indeed, the results of the present study showed that patients with DM had a higher rate of major amputations at 1 year and at 3 year and a lower amputation-free survival at 3 years in the propensity score adjusted analysis compared to non diabetic patients.

The proportion of women among patients with DM was higher than in patients without DM. One factor contributing to this discrepancy might be that women have smaller arteries than men. When adding the occlusive peripheral arteriosclerosis and atherosclerosis typical for patients with DM, there is therefore less margin in women than in men before development of ALLI. Treatment may also be more challenging in women with smaller atherosclerotic arteries, resulting in inferior leg salvage rate. After adjustment for confounders, there was also a trend towards higher major amputation rate at 3 years in women. In this context, it should be mentioned that improvement of glycaemic control is important to reduce the risk of peripheral arterial disease [12].The role of smaller vein diameter and narrower distal runoff arteries in women also seems to contribute to reduced graft patency [13].

Even though patients with DM were estimated to have less severe ischaemia according to Rutherford classification at admission, they had inferior leg survival rates after thrombolysis despite similar good revascularization. An explanation for these findings could be misclassification of patients with DM, since they presented themselves differently from patients without DM. Since peripheral neuropathy often is present among patients with DM [4], pain may be less expressed or even absent, leading to underestimation of severity of ischemia and inadequate drive and timing towards vascular imaging and leg revascularization compared to those without DM. There might also be issues on diagnostic assessments due to the higher proportion of renal insufficiency among diabetic patients making clinicians and radiologists less willing to perform immediate computed tomography angiography due to the risk of contrast induced renal failure and worsening of the patients´ already compromised renal function. In addition, the Rutherford classification [6] might be less applicable to patients with DM and ALLI, since sensory loss is a central finding when interpreting severity of ischemia in a patient with ALLI. The higher proportion of foot ulcer in diabetic patients might influence the physician in charge to underestimate the severity of ALLI and judge the ischemia as more chronic or acute-on-chronic rather than acute. As indicated in the present study, the presence of foot ulcer at admission was associated with higher major amputation rate at long-term in patients with DM compared to those with DM and no foot ulcer. The reason for this might be multiple, where secondary infection of foot ulcers plays a very important role, despite a successful revascularization [14].

Another striking difference was the absence of popliteal artery aneurysm in patients with DM, which not would have been detected without a comparative analysis. The pathogenesis of arterial aneurysms has been studied in patients with abdominal aortic aneurysm, and recent studies consistently report that DM protects an individual from developing abdominal aortic aneurysm [15, 16]. Since patients with popliteal artery aneurysm also to a greater extent develop other aneurysms in the aorta, iliac, and femoral arteries [17], it is highly likely that the same pathogenetic mechanisms are involved in formation of different arterial aneurysms. It has been proposed that glycated crosslinks in arterial tissue may act as a protective factor against aneurysm development [18]. Apart from pathogenetic considerations, the presence of popliteal artery aneurysm complicated by acute thromboembolism and ALLI has prognostic implications; inferior leg survival compared to other aetiologies such as thrombosis, embolism, or graft occlusion [19]. Major amputation rate was still increased in the DM group despite absence of popliteal artery aneurysm.

Assessment of the peripheral vascular tree has been performed in order to assess changes after attempt of thrombolysis [6]. In future studies it would also be worthwhile to categorize the peripheral arterial tree according to the updated TransAtlantic Inter-Society Consensus (TASC) classification [20] and/or ANGIO score [21] before and after leg revascularization, to analyse the influence of these tools on outcomes. The initial angiography in patients with ALLI undergoing thrombolysis should be scrutinized with the purpose to have an accurate diagnostic categorization. Emergent computed tomography angiography of the lower extremities has insufficient diagnostic accuracy due to interference of calcifications of the lower leg arteries [22], and duplex imaging or magnetic resonance imaging are seldom available in the emergency setting.

Limitations of the present study are attributed to its retrospective design. Patient selection bias is present since we do not know the overall number of patients with ALLI in the population. The number of patients undergoing open vascular surgery for ALLI during the study period was not documented either. However, in the present endovascular-oriented vascular center, a clear minority of ALLI patients are treated with open surgery. In a prospective study, it would have been worthwhile to include haemoglobin A1c (HbA1C), and specify DM as type 1 or 2, and also to specify the type of vascular surgery performed to obtain better understanding of differences between patients with and without DM, and between patients with type 1 and type 2 DM, respectively. Furthermore, assessment of patency was often done by clinical judgement only and not regularly based upon ultrasound examinations. This cohort study is based on a large patient sample, however, and confounding has been addressed by propensity score adjusted analysis.

## Conclusions

Patients with DM differ in clinical characteristics, presentation and aetiology in ALLI and have a higher rate of major amputation and lower amputation-free survival rate after intra-arterial thrombolysis compared to non-diabetics.

## References

[1] Taha AG, Byrne RM, Avgerinos ED, Marone LK, Makaroun MS, Chaer RA (2015). Comparative effectiveness of endovascular versus surgical revascularization for acute lower extremity ischemia. J Vasc Surg.

[2] Byrne RM, Taha AG, Avgerinos E, Marone LK, Makaroun MS, Chaer RA (2014). Contemporary outcomes of endovascular interventions for acute limb ischemia. J Vasc Surg.

[3] Graziani L, Silvestro A, Bertone V, Manara E, Andreini R, Sigala A, Mingardi R, De Giglio R (2007). Vascular involvement in diabetic subjects with ischemic foot ulcer: a new morphologic categorization of disease severity. Eur J Vasc Endovasc Surg.

[4] Claesson K, Kolbel T, Acosta S (2011). Role of endovascular intervention in patients with diabetic foot ulcer and concomitant peripheral arterial disease. Int Angiol.

[5] Kuoppala M, Åkesson J, Acosta S (2014). Outcome after thrombolysis for occluded endoprosthesis, bypasses and native arteries in patients with lower limb ischemia. Thromb Res.

[6] Rutherford RB, Baker JD, Ernst C, Johnston KW, Porter JM, Ahn S, Jones DN (1997). Recommended standards for reports dealing with lower extremity ischemia, revised version. J Vasc Surg.

[7] Earnshaw JJ, Whitman B, Foy C (2004). National audit of thrombolysis for acute leg ischemia (NATALI): clinical factors associated with early outcome. J Vasc Surg.

[8] Vittinghoff E, McCulloch C (2007). Relaxing the rule of ten events per variable in logistic and cox regression. Am J Epidemiol.

[9] Cepeda MS, Boston R, Farrar JT, Strom L (2003). Comparison of logistic regression versus propensity score when the number of events is low and there are multiple confounders. Am J Epidemiol.

[10] Martens EP, de Boer A, Pestman WR, Belitser SV, Stricker BH, Klungel OH (2008). Comparing treatment effects after adjustment with multivariable Cox proportional hazards regression and propensity score methods. Pharmacoepidemiol Drug Saf.

[11] Hinchliffe RJ, Brownrigg JRW, Andros G, Apelqvist J, Boyke E, Fitridge E, Mills JL, Reekers J, Shearman CP, Zierker E, Schaper NC (2016). Effectiveness of revascularization of the ulcerated foot in diabetes and peripheral arterial disease: a systematic review. Diabetes Metab Res Rev.

[12] Selvin E, Wattanakit K, Steffes MW, Coresh J, Sharett AR (2006). HbA1c and peripheral arterial disease in diabetes: the Atherosclerosis Risk in Communities study. Diabetes Care.

[13] Alpagut U, Ugurlucan M, Banach M, Mikhailidis DP, Dayioglu E (2008). Does gender influence the patency of infrainguinal bypass grafts?. Angiology.

[14] Elgzyri T, Larsson J, Nyberg F, Thörne J, Eriksson KF, Apelqvist J (2014). Early revascularization after admittance to a diabetic foot center affects the healing probability of ischemic foot ulcer in patients with diabetes. Eur J Vasc Endovasc Surg.

[15] Bhak RH, Wininger M, Johnson GR, Lederle FA, Messina LM, Ballard DJ, Wilson SE (2015). Factors associated with small abdominal aortic aneurysm expansion rate. JAMA Surg.

[16] Lederle FA, Noorbaloochi S, Nugent S, Taylor BC, Grill JP, Kohler TR, Cole L (2015). Multicentre study of abdominal aortic aneurysm measurement and enlargement. Br J Surg.

[17] Ravn H, Wanhainen A, Björck M (2008). Risk of new aneurysms after surgery for popliteal artery aneurysm. Br J Surg.

[18] Koole D, van Heerwaarden JA, Schalwijk CG, Lafeber FP, Vink A, Smeets MB, Pasterkamp G, Moll FL (2017). A potential role for glycated cross-links in abdominal aortic aneurysm disease. J Vasc Surg.

[19] Grip O, Kuoppala M, Acosta S, Wanhainen A, Åkeson J, Björck M (2014). Outcome and complications after intra-arterial thrombolysis for lower limb ischaemia with or without heparin infusion. Br J Surg.

[20] Jaff M, White C, Hiatt W, Fowkes G, Dormandy J, Razavi M, Reekers J, Norgren L (2015). An update on methods for revascularization and expansion of the TASC lesion classification to include below-the—knee arteries: a supplement to the Inter-Society consensus for the management of peripheral arterial disease (TASC II). J Endovasc Ther.

[21] Morris DR, Singh TP, Moxon JV, Smith A, Stewart F, Jones RE, Golledge J (2017). Assessment and validation of a novel angiographic scoring system for peripheral arterial disease. Br J Surg.

[22] Mishra A, Jain N, Bhagwat A (2017). CT angiography of peripheral arterial disease by 256-slice scanner: accuracy, advantages and disadvantages compared to digital subtraction angiography. Vasc Endovasc Surg.

